# Impact of Volatile Anesthetics on Oxidative Stress and Inflammation

**DOI:** 10.1155/2015/242709

**Published:** 2015-05-25

**Authors:** Yoon-Mi Lee, Byeng Chun Song, Kyung-Jin Yeum

**Affiliations:** Division of Food Bioscience, College of Biomedical & Health Sciences, Konkuk University, Chungwon-daero 268, Chungju-si, Chungcheongbuk-do 380-701, Republic of Korea

## Abstract

The safety of anesthesia, which is an important step for surgery, can be determined by its impact on oxidative stress and inflammation. The effects of volatile anesthetics such as isoflurane and sevoflurane on oxidative stress and inflammation are reviewed in various (1) cell lines, (2) rodents, and (3) human studies. Isoflurane and sevoflurane are reported to have antioxidant and anti-inflammatory effects in all cells with exception of neuronal cell lines. In addition, various animal studies have indicated that isoflurane and sevoflurane were not only safe but also reduced oxidative stress and inflammation in rodent models. In human studies, oxidative stress, inflammation, and DNA damage were not affected by isoflurane and sevoflurane in patients undergoing minor incision surgeries. On the other hand, elevated oxidative stress, inflammation, and DNA damage have been observed in patients undergoing major surgeries such as abdominal and orthopedic surgeries, hysterectomy, cholecystectomy, and thoracotomy. Although impact of anesthetics on oxidative stress and inflammation is still not clear due to the variations of patients' health conditions, types of surgery and the quantities of anesthetics, isoflurane, and sevoflurane can be considered safe anesthetics with respect to their effect on oxidative stress and inflammation in subjects undergoing minor surgery. Continuous effort evaluating the safety of anesthesia in various aspects is required.

## 1. Introduction

**Figure 1:**
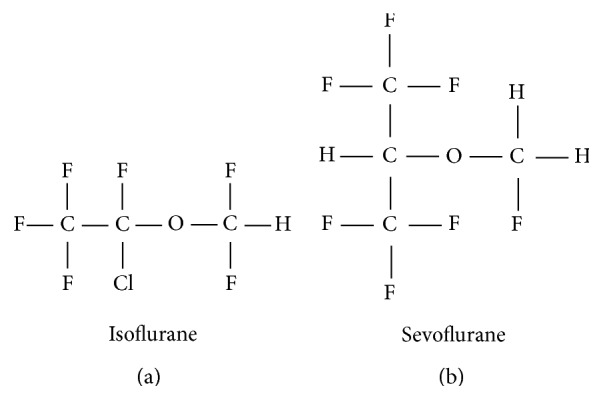
Structures of isoflurane and sevoflurane.

Anesthesia is an essential step for humans or animals undergoing surgery to provide analgesia. Anesthetic method can be classified by several mechanisms. Among various anesthesia, isoflurane [2-chloro-2-(difluoromethoxy)-1,1,1-trifluoro-ethane] and sevoflurane [fluoromethyl-2,2,2-trifluoro-1-(trifluoromethyl) ethyl ether] are the most widely used volatile anesthetics in clinical practice providing unconsciousness as well as analgesia (Figure 1). Isoflurane, which has been utilized since the 1980s, has a particularly low metabolism rate and solubility leading to reduced induction of anesthesia during surgery and shortened recovery time after surgery [1]. Sevoflurane began to be used a decade later and has a lower blood-gas partition coefficient than the other anesthetics leading to rapid induction of anesthesia and fast awakening after anesthesia [2, 3]. For several decades, the safety of anesthetics has drawn attention with respect to toxicity and potential side effects [4]. In this review, the impact of isoflurane and sevoflurane on oxidative stress and inflammation, which can be linked to prognosis of surgery, is discussed.

## 2. Oxidative Stress and Inflammation

Oxidative stress can be generated by an imbalance between the production of oxygen containing free radicals known as reactive oxygen species (ROS) and their elimination. Although ROS is essential for normal metabolism such as killing external harmful factors and maintaining cellular signaling in cells, overproduction of ROS can result in cellular dysfunction [5, 6]. Various enzymatic and nonenzymatic antioxidant systems contribute to the balance of ROS and have been studied for their protective effect on various chronic diseases [7, 8].

**Figure 2:**
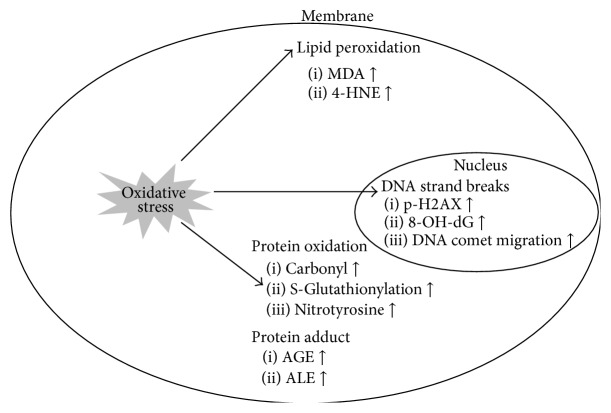
Biomarkers of oxidative damaged macromolecules. Oxidative stress leads to the damage of macromolecules such as DNA, lipid, and protein. The oxidative damaged macromolecules can be determined by their by-product under oxidative stress. MDA: malondialdehyde; 4-HNE: 4-4-hydroxy-2-nonenal; 8-OHdG: 8-hydroxydeoxyguanosine; AGE: advanced glycation end products; ALE: advanced lipoxidation end products.

The accumulation of oxidative stress plays an important role in the etiology of various chronic diseases such as neurodegenerative diseases, cardiac vascular diseases, and cancer [9–12]. Consequently, various biomarkers have been developed for determining oxidative stress status. For example, oxidative stress-induced DNA double strand breaks can be detected by phosphorylation of serine 139 residue of histone variant H2AX, upregulation of 8-hydroxydeoxyguanosine (8-OHdG), and migrated broken DNA by a comet assay (single-cell gel electrophoresis) [13–15]. Oxidative damaged lipids can be detected by the production of malondialdehyde (MDA) and 4-hydroxynoneal (4-HNE), which are well-known biomarkers for lipid peroxidation [16]. The oxidative stress also can produce protein carbonyls and cause modification of S-glutathionylation and nitrotyrosine [17]. The ROS levels in cells can be directly measured by fluorescence staining. The mechanism underlying measurement of stained cells is the conversion of dichlorofluorescin diacetate (DCFD-DA) to dichlorofluorescein (DCF) by oxidation [18]. Likewise, oxidative stress biomarkers (Figure 2) can be useful to predict the risk of oxidative stress associated chronic diseases.

The main purpose of inflammation is to protect the host from unfavorable stimuli such as pathogen infection and mechanical stress. However, the persistent inflammation by means of disruption of innate immunity or prolonged cellular stress-induced dysfunction can lead to an increase in the risk of chronic diseases [19]. Chronic diseases have a tendency of elevated inflammation by immune cell infiltration. Thus, inflammation is an attractive target for chronic diseases in the therapeutic point of view. In addition, oxidative stress has a major role in the incidence of chronic inflammation response by recruiting immune cells to the damaged areas [20]. In fact, oxidative stress has been reported to promote an activation of a variety of inflammation associated transcription factors such as nuclear factor-kappa B (NF-*κ*B), activator protein-1 (AP-1), p53, signal transducer and activator of transcriptions 3 (STAT3), hypoxia-inducible factor-1*α* (HIF-1*α*), and NF-E2 related factor-2 (Nrf2), followed by a production of a variety of inflammatory mediators aggravating inflammation response [21]. Also increased combinations of AGEs-RAGEs (receptor advanced glycosylation end products) can in turn induce inflammation [9]. Consequently, high amounts of inflammatory mediators by chronic inflammation cause oxidative stress [22].

**Figure 3:**
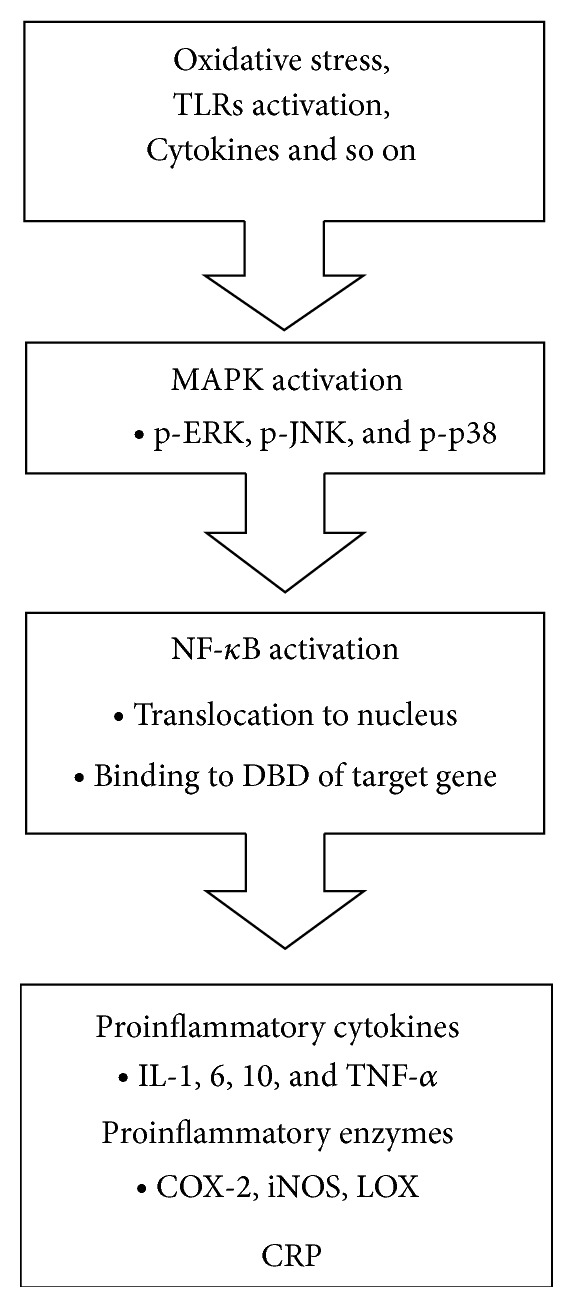
Activation of inflammatory signaling. Extracellular stimuli such as oxidative stress, TLRs activation, and infection lead to activating MAPK pathways by phosphorylation of ERK, JNK, and p38. Then, NF-*κ*B is translocated to the nucleus, which in turn binds to DBD of its target genes, proinflammatory cytokines, proinflammatory enzymes, and CRP. The target genes of NF-*κ*B are well known for aggravating inflammation responses. TLRs: toll-like receptors; ERK: extracellular signal-regulated kinases; JNK: c-Jun NH2-terminal; DBD: DNA binding domain; IL: interleukin; TNF-*α*: tumor necrosis factor-*α*; COX-2: cyclooxygeneases-2; iNOS: inducible nitric oxide synthase; LOX: lipoxygenase; CRP: C-reactive protein.

Among the various inflammation markers discovered, NF-*κ*B is the most important transcription factor in inflammation responses. NF-*κ*B, which is composed of p65 (RelA) and p50 heterodimers, normally exists as an inactive form by sequestering of inhibitor kappa B (I*κ*B) in the cytoplasm. Upon initiation of extracellular stimuli such as oxidative stress, toll-like receptors, various cytokines, and mitogen-activated protein (MAP) kinase pathways are activated. The MAPK pathways are classified into three subgroups; extracellular signal-regulated kinases (ERK), c-Jun NH2-terminal (JNK), and p38 protein kinases. Subsequently, the phosphorylated I*κ*B is ubiquitinated and degraded in the proteasome. The NF-*κ*B, which is free of I*κ*B, is translocated to the nucleus and functions as a transcription factor by binding its DNA binding domain. The NF-*κ*B promotes expression of proinflammatory cytokines (interleukins 1, 6, and 8 [IL-1, IL-6, IL-8] and tumor necrosis factor-*α* [TNF-*α*]), proinflammatory enzymes (cyclooxygeneases-2 [COX-2], inducible NO synthase [iNOS], and lipoxygenase [LOX]), and promoters of DNA damage [23, 24]. These target genes of NF-*κ*B aggravate inflammation response and produce ROS by these inflammatory mediators. The activation of inflammatory signaling is summarized in Figure 3. C-reactive protein (CRP) is another inflammation marker. It is produced in the liver and elevated in the early phases of inflammation. High levels of CRP or high sensitive CRP (hs-CRP) are released into the blood stream. It is regarded as a biomarker for cardiovascular diseases [25]. Currently, inflammatory cytokines and CRP levels in blood are the most commonly used biomarkers for inflammation in animals and humans. Inflammation can also be detected by upregulation of genes encoding inflammation in animal tissues and cells.

**Figure 4:**
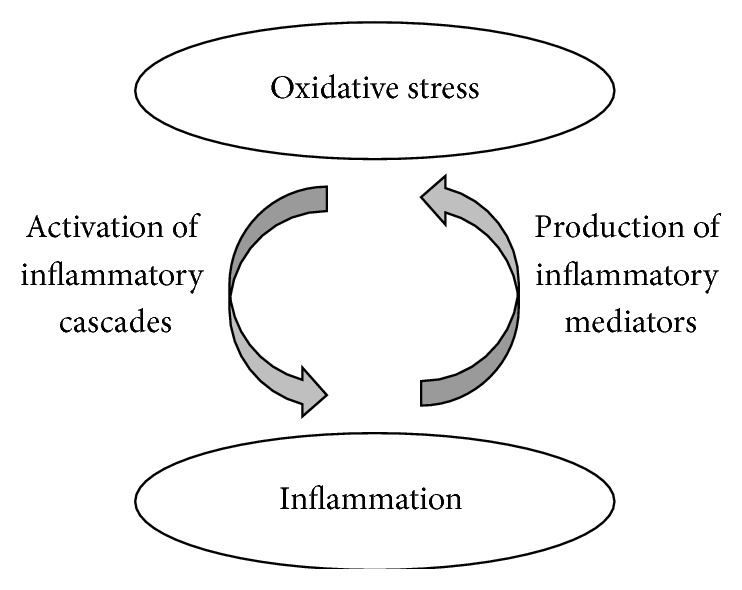
Interrelation of oxidative stress and inflammation. Oxidative stress-induced inflammatory cascades cause the inflammation, which in turn increases oxidative stress.

Notably oxidative stress and inflammation are interrelated as shown in Figure 4 and associated with anesthetics. The following sections discuss research studies with respect to the effect of volatile anesthetics, isoflurane, and sevoflurane on oxidative stress and inflammation in various cell lines, animal models, and humans.

## 3. The Effects of Isoflurane and Sevoflurane on Oxidative Stress and Inflammation in Cells

Studies on the effects of volatile anesthetics on oxidative stress and inflammation on various cell lines are summarized in Table 1. The immunomodulatory effects of volatile anesthetics on cells have been studied for several decades [26]. During or after surgery, inflammation in the lung, heart, or brain is a severe health problem leading to various complications and recurrences. Therefore,* in vitro* inflammation studies on isoflurane and sevoflurane were mainly conducted in the lung and brain cells. Sepsis is a commonly suffered inflammatory disease after surgery with a high rate of mortality.* In vitro* model of sepsis is established by lipopolysaccharide (LPS) treatment in human umbilical vein endothelial cells (HUVEC). The postconditioning of sevoflurane by various doses (0%, 1%, 3%, or 7%) for 1 hour in* in vitro* sepsis environment leads to an increase of cell viability and decrease of inflammatory indicators such as toll-like receptor (TLR) 2, toll-like receptor (TLR) 4, and cytokines, such as TNF-*α* and IL-6 [27]. When sevoflurane (2.2%) was added to LPS-stimulated alveolar epithelial cells, sevoflurane was reported to attenuate immune function expressed by neutrophil chemoattractant-1, monocyte chemoattractant protein-1, and intracellular adhesion molecule-1 as well as inflammatory enzyme inducible nitric oxide synthase (iNOS) [28]. In addition, isoflurane (0.7%) is reported to reduce inflammatory cascades such as p38 MAPK, NF-*κ*B DNA binding affinity, and its transcriptional activities expressed by proinflammatory cytokines, chemokines, and COX-2 in the zymosan-induced inflammation in murine macrophage cells (murine Kupffer cells). It was suggested that the isoflurane inhibited ROS production, which in turn blocks the inflammatory cascades [18]. Furthermore, Boost et al. demonstrated that 1 MAC (minimal alveolar concentration) of sevoflurane and isoflurane suppressed inflammation response in human monocytic THP-1 cells* via* decreasing inflammatory cascades. Under the treatment of sevoflurane and isoflurane on TNF-*α*-stimulated THP-1 cells, nuclear translocation of NF-*κ*B was blocked and still remains with I*κ*B in the cytoplasm [29] resulting in limited production of inflammatory cytokines.

Postoperative cognitive dysfunction has been associated with neurodegenerative disease. Thus, various studies have been conducted to determine the effect of anesthetics on neuron cells. Microglial cells are macrophage existing in central nervous system, and inflammation can be induced by LPS. However, sevoflurane (2%, 4%) and isoflurane (1.2%, 2.4%) are reported to have no positive effect on neuroinflammation determined by cytokine levels [30]. In addition, isoflurane (1.2 MAC) exacerbated neuronal death in oxygen-glucose deprivation-exposed SH-SY5Y neuroblastoma cells as well as increased NF-*κ*B transcriptional activities [31]. Taken together, isoflurane and sevoflurane are reported to have anti-inflammatory effects on inflammatory-stimulated lung and immune cells but not on brain cells. Thus, usage of anesthetics in patients with neuronal injury should be considered with prudence.

## 4. The Effect of Isoflurane and Sevoflurane on Oxidative Stress and Inflammation in Animal Models

Various studies determining the effects of isoflurane and sevoflurane on oxidative stress and inflammation in rodent models are presented in Table 2. Animal experiments using isoflurane and sevoflurane are mainly performed in cardiac and lung diseases models. Isoflurane has a protective effect on rat ventricular myocyte against the imbalance of oxygen states such as hypoxia, treatment of hydrogen peroxide, and neutrophil-induced inflammation responses [32]. It was also found that preconditionings of sevoflurane and isoflurane have beneficial effects on rat ischemia model. The inflammatory mediators and oxidative-damage markers were decreased by sevoflurane and isoflurane, respectively, while both volatile anesthetics increased antioxidant enzymes. Furthermore, the volatile anesthetics (2% sevoflurane, 1.5% isoflurane) had promising effect on relieving ischemia via regulating apoptosis-related genes, which in turn reduced apoptosis [33]. Among various comorbidities in diabetic patients, coronary heart diseases is linked with stroke and early death. Interestingly, isoflurane (up to 3%) was reported to decrease myocardial contraction and oxidative stress, the common symptoms of coronary heart diseases, in Zucker diabetic fatty rats [34]. When general inflammation was induced by Zymosan in mice, a subanesthetic dose of isoflurane (0.7%) increased the survival rate from 10% to 45% and reduced inflammation by upregulating antioxidant enzymes such as superoxide dismutase and catalase in blood and lung tissue. It is also demonstrated that the protective effects of isoflurane can be altered by the treatment of catalase inhibitor [35]. In another zymosan-induced inflammation model of mice, 1.4% isoflurane reduced the levels of IL-1*β*, IL-6, TNF-*α*, macrophage inflammatory protein 2 (MIP2), iNOS, and nuclear NF-*κ*B. Moreover, apoptosis was inhibited in the isoflurane-treated mice [36]. LPS is also widely used for inducing general inflammation in animal models. Among the rats injected with LPS from* Escherichia coli*, rats receiving sevoflurane (1 MAC) had significantly lower TNF-*α*, IL-1*β*, and IL-10 cytokine levels as compared to those of LPS-only treated rats [37]. Isoflurane (1.4%) pretreatment was also reported to suppress LPS-induced TNF-*α* cytokine levels [38]. In the sepsis model of cecal ligation and puncture (CLP) in mice, sevoflurane (1.2 MAC) was reported to have immunomodulatory effects by decreasing IL-6, monocyte chemoattractant protin-1 (MCP-1), and increased survival rates up to 83% [39]. In addition, in a study of CLP-induced sepsis model in rats, pretreatment of 2% sevoflurane and 1.5% isoflurane was found to be significantly enhancing survival rate of rats in the seventh day after CLP by 75% and 38%, respectively. When the plasma inflammatory mediators and biomarkers of oxidative stress in the lung were determined before and after CLP using sevoflurane and isoflurane, plasma cytokines such as TNF-*α*, IL-6, and IL-1*β* were significantly reduced by the treatment of sevoflurane or isoflurane. Furthermore, the nitric oxide (NO) and MDA levels were decreased and total antioxidant capacity (TAC) was increased by the treatment of volatile anesthetics. In this study, sevoflurane had more effect on antioxidant capacity and anti-inflammatory activity [40]. In case of liver transplantation experimental model, anesthesia with sevoflurane provided antioxidant effects by attenuating serum lipid peroxidation determined by thiobarbituric acid reactive substances (TBARS) [41]. As described, various animal studies indicated that isoflurane and sevoflurane were not only safe but also reduced the oxidative stress and inflammation in animal models.

## 5. The Effect of Isoflurane and Sevoflurane on Oxidative Stress and Inflammation in Humans

Although various studies have been conducted to determine the effect of isoflurane and sevoflurane on oxidative stress and inflammation for several decades, the results are still controversial probably due to the variations of patients' health conditions and types of surgery. Studies on the effects of volatile anesthetics on oxidative stress and inflammation in humans are summarized in Table 3.

In the past, many studies mainly dealt with the negative effects of anesthetics on oxidative stress and inflammation in patients who experienced serious surgery. Increased lipid and protein oxidations after surgery using sevoflurane and isoflurane has been reported to be a biomarker of nephrotoxicity [42]. A study compared the DNA damage determined by a comet assay between patients-received isoflurane (1 to 1.5%) and sevoflurane (1 to 1.5%) for an abdominal surgery and found an increased comet tail migration at 120 min after anesthesia. Isoflurane and sevoflurane were reported to have similar effects on DNA damage [43]. The peripheral lymphocyte in patients exposed sevoflurane (2%) in order to get orthopedic surgery had also an increased DNA damage and decreased glutathione contents, which regulates redox state by scavenging ROS [44]. Likewise, isoflurane-received patients undergoing abdominal surgery to extract organ such as uterus and gallbladder were reported to release higher amounts of cytokines than intravenous anesthesia-received patients undergoing same surgery [45, 46]. Sevoflurane (6 to 8%) was reported to aggravate various pulmonary functions via a release of inflammatory factors in lung cancer patients who resected a portion of their lung with one-lung ventilation [47].

On the other hand, recent growing evidence indicated that sevoflurane (1.9%) and isoflurane (1.2%) did not affect DNA damage determined by a comet assay during and after anesthesia for minimally invasive surgery [48]. Peripheral lymphocyte was isolated in patients exposed to isoflurane (1.2%) for a minor electric surgery. Interestingly isoflurane did not affect any DNA damage in lymphocyte of patients, indicating that isoflurane has no genotoxicity or cytotoxicity [49]. Moreover, sevoflurane represented antioxidant activity determined by MDA and antioxidant enzyme levels in erythrocyte of patients for abdominal surgery [50]. Balanced anesthesia using isoflurane (1.0 to 1.5 MAC) also did not trigger oxidative damage and DNA oxidation in patients undergoing minimally invasive surgery. Isoflurane did not change IL-6 cytokine levels as well in this study [51]. Sevoflurane (1.9%) was also reported to be safe not causing any change in DNA damage and lipid peroxidation [52].

Recent studies on anti-inflammatory effects of sevoflurane and patients-receiving sevoflurane (1 MAC) were reported to inhibit pulmonary inflammatory cytokines such as TNF-*α*, IL-1*β*, IL-6, and IL-8 [53]. Another study also performed the effect of sevoflurane on inflammation in patients undergoing thoracic surgery with one-lung ventilation. TNF-*α*, IL-1*β*, IL-6, IL-8, and monocyte chemoattractant protein (MCP1) were measured in blood samples of the patients. They found that sevoflurane (1 MAC) attenuated inflammatory response after one-lung ventilation compared to intravenous anesthetics propofol [54]. In addition, the inflammation levels do not seem to be affected by the sevoflurane (1 to 1.5 MAC) treatment in patients. The cytokine levels such as TNF-*α*, IL-1*β*, IL-2, IL-4, IL-6, IL-7, IL-10, IL-12, and IFN-*γ* were not affected by sevoflurane during operation period except for IL-6 on the next day of operation due to surgical stress in patients with minimally invasive surgery [55]. It is interesting to note that sevoflurane does not alter DNA damage in operating room personnel [56]. On the other hand, doctors who are exposed to waste anesthetic gases such as isoflurane and sevoflurane during surgery for up to a 22 weeks residency period had increased oxidative stress indicating a need of an appropriate system to protect doctors from this noxious condition [57].

## 6. Conclusion and Future Directions

The safety of anesthesia for surgery has been an important concern for decades. Here we reviewed the impact of isoflurane and sevoflurane on oxidative stress and inflammation in rodent and human cells, rodent models, and humans. In case of cells and animals, various experiments attempted to evaluate oxidative stress and inflammation under isoflurane or sevoflurane exposure. Accordingly, disease models have been established in lung, cardiac, and brain. Isoflurane and sevoflurane had antioxidant and anti-inflammatory effects on various cells except for neuronal cells. In contrast to the past studies indicating elevated oxidative stress, inflammation, and DNA damage by isoflurane and sevoflurane in patients undergoing major surgery, a growing body of recent studies indicates a safety of isoflurane and sevoflurane in patients undergoing minor incision surgery. Although there is still uncertainty existing due to the variations of patient's status, type of surgery, and administered amount of anesthetics, isoflurane and sevoflurane are considered safe with respect to oxidative stress and inflammation for patients undergoing minor surgery. Further study warrants evaluating the safety of anesthesia in various other aspects.

## Figures and Tables

**Table 1:** Effects of volatile anesthetics on oxidative stress and inflammation in cells.

Disease model	Stimulus	Cells	Anesthetics	Effect	Reference
Sepsis	LPS	HUVEC (human)	Sevoflurane	Anti-inflammatory effects	[27]

Acute lung injury	LPS	AEC (rat)	Sevoflurane	Anti-inflammatory effects	[28]

MODS	Zymosan	Kupffer cells (murine)	Isoflurane	Antioxidant effects	[18]
				Anti-inflammatory effects	

Inflammation	TNF-*α*	Monocytic THP-1 (human)	Sevoflurane, isoflurane	Anti-inflammatory effects	[29]

POCD	LPS	Microglial BV-2 (murine)	Sevoflurane, isoflurane	No effect on cytokine levels	[30]

Neuron injury	OGD	SH-SY5Y (human)	Isoflurane	Increased neuronal cell death	[31]

LPS, lipopolysaccharides; HUVEC, human vascular endothelial cells; AEC, alveolar epithelial cells; MODS, multiple organ dysfunction syndrome; TNF-*α*, tumor necrosis factor-*α*, POCD, postoperative cognitive dysfunction; OGD, oxygen-glucose deprivation.

**Table 2:** Effects of volatile anesthetics on oxidative stress and inflammation in rodents.

Disease model	Inducer	Anesthetics	Effects	Reference
Cardiac ischemia in rat myocyte	Hypoxia	Isoflurane	Antioxidant effects	[32]
	Hydrogen peroxide		Antiapoptosis effects	
	Neutrophil		Cardioprotective effects	

Cerebral ischemia in rats	Cerebral artery occlusion	Sevoflurane, isoflurane	Antioxidant effects	[33]
			Anti-inflammatory effects	

Diabetic rats	Mutation in the leptin receptor gene	Isoflurane	Antioxidant effects	[34]
			Decreased myocardial contraction	

MODS in mice	Zymosan	Isoflurane	Anti-inflammatory effects	[18]

MODS in mice	Zymosan	Isoflurane	Anti-inflammatory effects	[35]

MODS in mice	Zymosan	Isoflurane	Antiapoptosis effects	[36]
			Anti-inflammatory effects	

Inflammation in rats	LPS	Sevoflurane	Anti-inflammatory effects	[37]

Inflammation in rats	LPS	Isoflurane	Anti-inflammatory effects	[38]

Sepsis in mice	Cecal ligation and puncture	Sevoflurane	Anti-inflammatory effects	[39]

Sepsis in rat	Cecal ligation and puncture	Sevoflurane, isoflurane	Anti-inflammatory effects	[40]

Rat liver transplantation	—	Sevoflurane	Antioxidant effects	[41]

MODS, multiple organ dysfunction syndrome; LPS, lipopolysaccharide.

**Table 3:** Effects of volatile anesthetics on oxidative stress and inflammation in humans.

Subject of study	Anesthetics	Effects	References
Patients (elective lower abdominal surgery)	Isoflurane, sevoflurane	Increased DNA damage	[43]

Patients (orthopedic surgery)	Sevoflurane	Increased DNA damage	[44]

Patients (elective hysterectomy)	Isoflurane	Increased inflammation response	[45]

Patients (elective cholecystectomy)	Isoflurane	Increased inflammation response	[46]

Patients (elective thoracotomy lobectomy)	Sevoflurane	Pulmonary dysfunction by increased inflammation response	[47]

Patients (otorhinological surgery)	Isoflurane, sevoflurane	No alteration of DNA damage	[48]

Patients (otorhinological surgery)	Isoflurane	No alteration of DNA damage	[49]

Patients (abdominal surgery)	Sevoflurane	Antioxidant activity	[50]

Patients (otorhinological surgery)	Isoflurane	No induction of oxidative stress	[51]
		No induction of inflammation	

Patients (otorhinological surgery)	Sevoflurane	No alteration of redox state	[52]

Patients (open thoracic surgery)	Sevoflurane	Anti-inflammatory effects	[53]

Patients (open thoracic surgery)	Sevoflurane	Anti-inflammatory effects	[54]

Patients (otorhinological surgery)	Isoflurane, sevoflurane	No alteration of cytokine profiles	[55]

Operating room personnel	Sevoflurane	No alteration of DNA damage	[56]

Operating room personnel	Isoflurane	Increased DNA damage	[57]
